# Clinical Evaluation of ^68^Ga-FAPI-RGD for Imaging of Fibroblast Activation Protein and Integrin α_v_β_3_ in Various Cancer Types

**DOI:** 10.2967/jnumed.122.265383

**Published:** 2023-08

**Authors:** Liang Zhao, Xuejun Wen, Weizhi Xu, Yizhen Pang, Long Sun, Xiaoming Wu, Pengfei Xu, Jingjing Zhang, Zhide Guo, Qin Lin, Xiaoyuan Chen, Haojun Chen

**Affiliations:** 1 Department of Nuclear Medicine and Minnan PET Center, Xiamen Cancer Center, First Affiliated Hospital of Xiamen University, School of Medicine, Xiamen University, Xiamen, China;; 2 Department of Radiation Oncology, Xiamen Key Laboratory of Radiation Oncology, Xiamen Cancer Center, First Affiliated Hospital of Xiamen University, School of Medicine, Xiamen University, Xiamen, China;; 3 Departments of Diagnostic Radiology, Surgery, Chemical and Biomolecular Engineering, and Biomedical Engineering, Yong Loo Lin School of Medicine and Faculty of Engineering, National University of Singapore, Singapore;; 4 Clinical Imaging Research Centre, Centre for Translational Medicine, Yong Loo Lin School of Medicine, National University of Singapore, Singapore;; 5 State Key Laboratory of Molecular Vaccinology and Molecular Diagnostics and Center for Molecular Imaging and Translational Medicine, School of Public Health, Xiamen University, Xiamen, China;; 6 College of Nuclear Science and Technology, Harbin Engineering University, Harbin, China; and; 7 Nanomedicine Translational Research Program, NUS Center for Nanomedicine, Yong Loo Lin School of Medicine, National University of Singapore, Singapore

**Keywords:** fibroblast activation protein, integrin α_v_β_3_, heterodimer, cancer-associated-fibroblasts, PET

## Abstract

Radiolabeled fibroblast activation protein (FAP) inhibitors (FAPIs) and Arg-Gly-Asp (RGD) peptides have been extensively investigated for imaging of FAP- and integrin α_v_β_3_–positive tumors. In this study, a FAPI-RGD heterodimer was radiolabeled with ^68^Ga and evaluated in patients with cancer. We hypothesized that the heterodimer, recognizing both FAP and integrin α_v_β_3_, would be advantageous because of its dual-receptor–targeting property. **Methods:** The effective dose of ^68^Ga-FAPI-RGD was evaluated in 3 healthy volunteers. The clinical feasibility of ^68^Ga-FAPI-RGD PET/CT was evaluated in 22 patients with various types of cancer, and the results were compared with those of ^18^F-FDG and ^68^Ga-FAPI-46. **Results:** ^68^Ga-FAPI-RGD was tolerated well, with no adverse events in any of the healthy volunteers or patients. The effective dose from ^68^Ga-FAPI-RGD PET/CT was 1.01 × 10^−2^ mSv/MBq. In clinical investigations with different types of cancer, the radiotracer uptake and tumor-to-background ratio (TBR) of primary and metastatic lesions in ^68^Ga-FAPI-RGD PET/CT were significantly higher than those in ^18^F-FDG PET/CT (primary tumors: SUV_max_, 18.0 vs. 9.1 [*P* < 0.001], and TBR, 15.2 vs. 5.5 [*P* < 0.001]; lymph node metastases: SUV_max_, 12.1 vs. 6.1 [*P* < 0.001], and TBR, 13.3 vs. 4.1 [*P* < 0.001]), resulting in an improved lesion detection rate and tumor delineation, particularly for the diagnosis of lymph node (99% vs. 91%) and bone (100% vs. 80%) metastases. ^68^Ga-FAPI-RGD PET/CT also yielded a higher radiotracer uptake and TBR than ^68^Ga-FAPI-46 PET/CT did. **Conclusion:** ^68^Ga-FAPI-RGD exhibited improved tumor uptake and TBR compared with ^18^F-FDG and ^68^Ga-FAPI PET/CT. This study demonstrated the safety and clinical feasibility of ^68^Ga-FAPI-RGD PET/CT for imaging of various types of cancer.

## Visual Abstract

**Figure:**
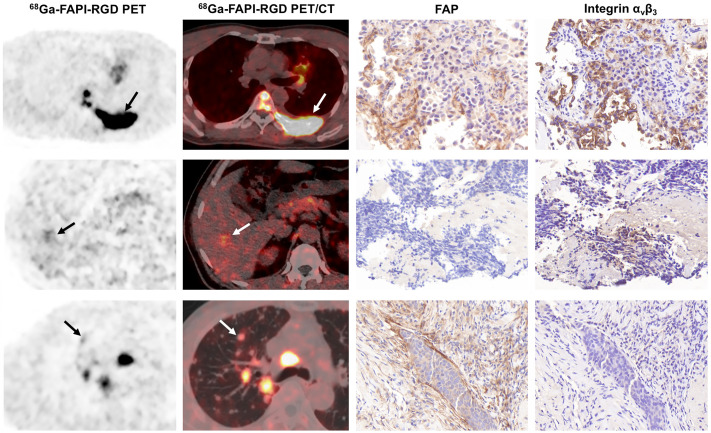

Tumor receptor imaging is an important component of oncologic molecular imaging and plays a key role in cancer diagnosis and management. It is made possible by the intense expression of specific biomarkers in the cellular membrane. Integrin α_v_β_3_ is a heterodimeric transmembrane glycoprotein that is highly expressed in activated endothelial cells, newly formed blood vessels, and several types of tumor cells. In contrast, it exhibits low or no expression in normal cells (1). Therefore, integrin α_v_β_3_ is a promising target for tumor imaging and therapy (2). The tripeptide Arg-Gly-Asp (RGD) moiety exhibits a high binding affinity and specificity for integrin α_v_β_3_. Various RGD-based cyclic peptides have been labeled with radionuclides and extensively evaluated for PET or SPECT imaging of cancers (2). However, the rapid blood clearance of RGD peptides is one of the reasons that their application is limited in radionuclide therapy (3). The need to improve the pharmacokinetics of RGD peptides has led to multimeric strategies or conjugation with albumin-binding moieties (3*,*^4^). These approaches allow the transformation of a drug into a theranostic agent for use as both an imaging agent and a therapeutic agent.

The importance of the tumor microenvironment in cancer development and clinical prognosis is now widely appreciated (5). Besides tumor cells and tumor angiogenesis, cancer-associated fibroblasts of the tumor microenvironment are the major components of solid tumors (6). Fibroblast activation protein (FAP)–α is highly expressed in cancer-associated fibroblasts in most epithelial cancers but is weakly expressed in normal tissues, making it an attractive target for cancer imaging and therapy (7). In the past few years, quinoline-based FAP inhibitor (FAPI) PET/CT has become an active field in nuclear oncology (7*,*^8^). Various studies have demonstrated that FAPI-based radiopharmaceuticals are promising radiotracers for cancer diagnosis, staging, and restaging. They may be better alternatives for cancer types that exhibit low to moderate uptake of ^18^F-FDG, including gastric, pancreatic, and liver cancers (9).

Recently, a heterodimeric peptide, denoted as FAPI-RGD, was synthesized from FAPI-02 and cyclo-RGD-d-Phe-Lys (c[RGDfK]) for targeting both FAP and integrin α_v_β_3_ receptors (10). It was radiolabeled with ^68^Ga for preclinical evaluation and was further assessed in a pilot clinical study. The tumor uptake and retention of ^68^Ga-FAPI-RGD were significantly greater than those of ^68^Ga-FAPI-46 and ^68^Ga-c(RGDfK) in mouse xenografts (10). Furthermore, this pilot clinical study on 6 patients demonstrated that ^68^Ga-FAPI-RGD PET/CT enabled visualization of tumor lesions with favorable imaging contrast. These results encouraged us to further explore the role of ^68^Ga-FAPI-RGD PET/CT for cancer imaging.

The aim of this study was to evaluate, in patients with various types of cancer, the radiotracer uptake and clinical feasibility of ^68^Ga-FAPI-RGD PET/CT compared with those of ^18^F-FDG and ^68^Ga-FAPI-46 PET/CT.

## MATERIALS AND METHODS

### Chemistry and Radiochemistry

The detailed synthesis procedure for FAPI-RGD was reported recently (10). Information on the chemicals and reagents is briefly presented in the supplemental materials (available at http://jnm.snmjournals.org). ^68^Ga-radiolabeling of FAPI-02, FAPI-46, c(RGDfK), and FAPI-RGD variants was performed as previously described (11–13). In brief, 25 nmol of FAPI-RGD in 1 mL of sodium acetate buffer (0.25 M) were reacted with 4 mL of ^68^Ga-solution (1.1 GBq in 0.6 M HCl) at 100°C for 15 min. For clinical imaging, the final product was passed through a 0.22-μm Millipore filter for sterilization of each preparation of ^68^Ga-FAPI-RGD. Quality control was performed using ultraviolet and radio-high-performance liquid chromatography (supplemental materials). The stability of the radiolabeled compound was determined by incubating the product in phosphate-buffered saline at 37°C and analyzing it via radio-high-performance liquid chromatography after 1, 2, and 4 h of incubation.

### Clinical PET/CT Imaging in Healthy Volunteers and Patients with Cancer

The prospective clinical study protocol was approved by the institutional review board of the First Affiliated Hospital of Xiamen University and was registered at ClinicalTrials.gov (NCT05543317). Written informed consent was obtained from all healthy volunteers and patients. The dose of intravenously injected ^68^Ga-FAPI-RGD was calculated according to the participants’ body weight (3.0–3.7 MBq/kg), which corresponds to approximately 7–8 nmol per subject. Safety data (blood pressure, heart rate, and temperature) and adverse events were recorded before and 4 h after injection of ^68^Ga-FAPI-RGD. The PET/CT scanning and reconstruction protocols are presented in the supplemental materials. For dosimetry evaluation, ^68^Ga-FAPI-RGD PET imaging was performed at 30, 60, and 180 min after tracer injection. Time–activity curve fitting and subsequent dose calculations were performed using OLINDA/EXM software, version 1.1 (14).

All patients underwent paired ^68^Ga-FAPI-RGD and ^18^F-FDG PET/CT scans. An additional ^68^Ga-FAPI-46 PET/CT scan was performed for comparative purposes depending on the patient’s willingness. We evaluated the in vivo distribution pattern of ^68^Ga-FAPI-RGD at later time points, by performing 3-h-delayed ^68^Ga-FAPI-RGD PET/CT scans for all patients (because of the relatively short half-life [68 min] of ^68^Ga). All PET images were evaluated by 2 board-certified and experienced nuclear medicine physicians. Disagreements were resolved via consensus. For quantitative analysis, the SUV_max_ was used to quantify radiopharmaceutical uptake by normal organs and tumor tissues. Tracer uptake in normal organs (background) was quantified by SUV_mean_, which was delineated with a sphere that had a diameter of 1 cm (for small organs, including thyroid, salivary gland, and pancreas) to 2 cm (for other organs, including brain, heart, liver, kidney, spleen, muscle, and bone marrow) placed inside the organ parenchyma. The tumor-to-background ratio (TBR) was calculated as tumor SUV_max_/background SUV_mean_.

To evaluate the diagnostic performance of ^68^Ga-FAPI-RGD and ^18^F-FDG PET imaging, the results of the visually interpreted PET images were compared with the histopathologic results (via surgery or biopsy), which were used as the gold standard for the final diagnosis. For patients for whom tissue diagnosis was not applicable, clinical and radiographic follow-up data were used as the reference standard to validate the PET/CT findings. Lesions were considered malignant on the basis of any of the following follow-up criteria: typical malignant features confirmed by multimodal medical imaging, significant progression on follow-up imaging, or a significant decrease in posttreatment tumor size. The minimum follow-up period was 3 mo. Histopathologic staining of surgical and biopsy samples was performed as previously described (15).

### Statistical Analysis

All statistical analyses were conducted using Prism (version 8.0; GraphPad Software Inc.) and SPSS Statistics (version 22.0; IBM Corp.). All quantitative data are expressed as the mean. The Wilcoxon matched-pairs signed-rank test was used to compare SUVs derived from ^18^F-FDG, ^68^Ga-FAPI-RGD, and ^68^Ga-FAPI-46 PET/CT. The McNemar test was used to compare the lesion detectability of the different PET/CT scans. Statistical significance was defined as a *P* value of less than 0.05.

## RESULTS

### Synthesis and Radiolabeling

^68^Ga-FAPI-RGD was radiolabeled at a specific activity of approximately 33 GBq/μmol, with over 99% radiochemical purity after purification (Supplemental Fig. 1A). High-performance liquid chromatography analysis revealed that ^68^Ga-FAPI-RGD had a high stability for up to 4 h, with no significant demetallation observed in phosphate-buffered saline (>99%) (Supplemental Figs. 1B–1D).

### Safety and Radiation Dosimetry in Healthy Volunteers

**FIGURE 1.**
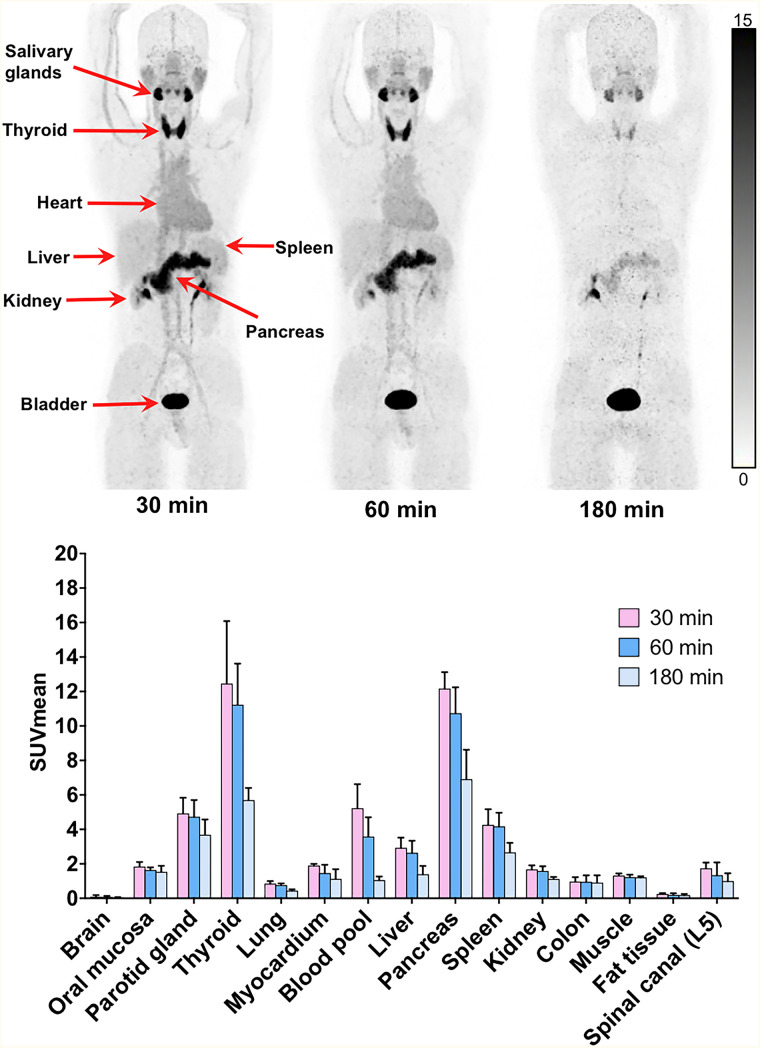
^68^Ga-FAPI-RGD at 30, 60, and 180 min after injection in healthy volunteers, and SUV_mean_ of normal organs at corresponding time points (*n* = 3).

All observed vital signs (including temperature, heart rate, and blood pressure) remained normal during the injection and 4-h postinjection follow-up. ^68^Ga-FAPI-RGD was tolerated well, with no adverse events in any of the healthy volunteers or patients. Representative PET images and biodistribution data of healthy volunteers (*n* = 3) are provided in Figure 1. Tracer uptake rapidly decreased in most normal organs from 30 to 180 min, particularly in the thyroid, pancreas, and salivary glands. The ^68^Ga-FAPI-RGD effective dose was 1.01 × 10^−2^ mSv/MBq (Supplemental Table 1), which was comparable to that of ^68^Ga-FAPI-02 (1.80 × 10^−2^ mSv/MBq) (16). The organ with the highest effective dose was the thyroid (3.01 × 10^−3^ mSv/MBq), followed by the urinary bladder wall (1.37 × 10^−3^ mSv/MBq), liver (1.10 × 10^−3^ mSv/MBq), and lungs (1.09 × 10^−3^ mSv/MBq).

### Patients’ Characteristics

From July 1 to September 15, 2022, 22 patients with cancer (15 men; median age, 57 y; range, 34–79 y; 17 for staging and 5 for restaging) who underwent paired ^68^Ga-FAPI-RGD and ^18^F-FDG PET/CT were enrolled in this study. The median interval between the 2 scans was 4 d (range, 1–7 d). Furthermore, 7 of the 22 patients underwent additional ^68^Ga-FAPI-46 PET/CT for comparison. Among the 22 patients, the final diagnosis was based on the histopathologic results for 20 and diagnostic radiology results for 2. Detailed information on the enrolled patients is provided in Supplemental Table 2.

### Dual-Time-Point ^68^Ga-FAPI-RGD PET/CT Imaging in Patients with Cancer

**FIGURE 2.**
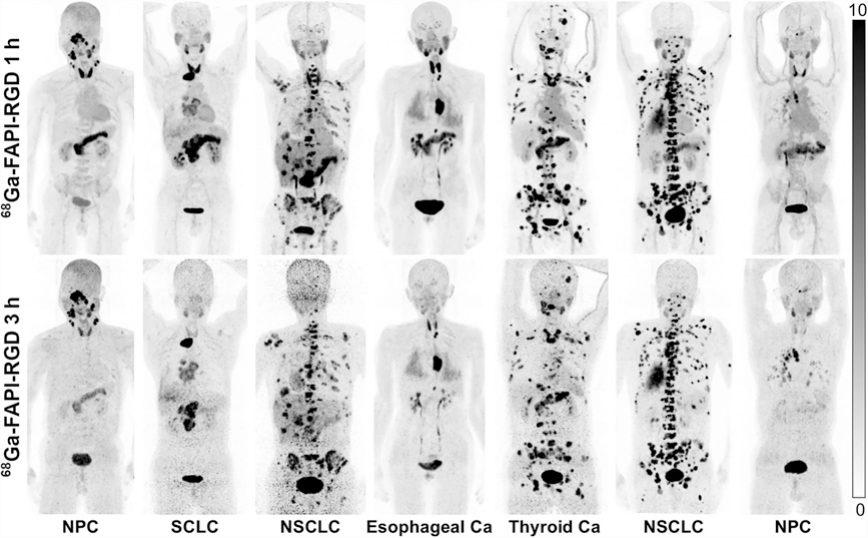
Maximum-intensity-projection images of ^68^Ga-FAPI-RGD PET/CT at 1 and 3 h after injection in different types of cancer. Ca = cancer; NPC = nasopharyngeal carcinoma; SCLC = small cell lung cancer.

To evaluate the in vivo distribution pattern of the radiotracer and tumor uptake over time, dual-time-point ^68^Ga-FAPI-RGD PET/CT (1 vs. 3 h) was performed for all patients. As demonstrated in Figure 2, most tumor lesions demonstrated increased uptake over time. Specifically, SUV_max_ derived from the delayed scan (3 h) was significantly higher than SUV_max_ derived from routine scans (1 h) in the primary tumors (18.0 vs. 12.6; *P* < 0.001), lymph node metastases (12.1 vs. 9.3; *P* < 0.001), lung metastases (median, 8.0 vs. 4.6; *P* < 0.001), and bone metastases (16.2 vs. 13.2; *P* < 0.001). Interestingly, background activity decreased greatly over time. Consequently, TBR improved significantly in primary tumors with involved lymph nodes and with lung, liver, peritoneal, and bone metastases. Detailed data are presented in Supplemental Table 3.

### Comparison of ^68^Ga-FAPI-RGD and ^18^F-FDG Uptake in Patients with Cancer

**FIGURE 3.**
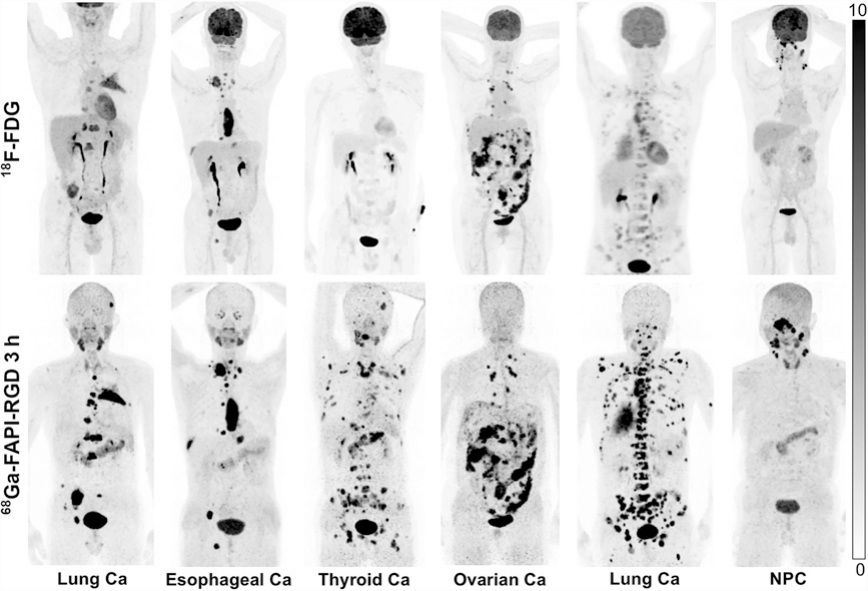
Maximum-intensity-projection images of ^18^F-FDG and ^68^Ga-FAPI-RGD PET/CT in patients with different types of cancer. Ca = cancer; NPC = nasopharyngeal carcinoma.

Among the 17 patients who underwent paired ^68^Ga-FAPI-RGD and ^18^F-FDG PET/CT for initial staging, ^68^Ga-FAPI-RGD PET/CT allowed detection of all primary tumors (19/19) with intense radiotracer uptake, whereas ^18^F-FDG PET/CT resulted in 3 missed tumor lesions in 1 patient with multifocal breast cancer. In all primary tumors, SUV_max_ was significantly higher when derived from ^68^Ga-FAPI-RGD PET/CT than from ^18^F-FDG (18.0 vs. 9.1, *P* < 0.001). Furthermore, the TBRs of the primary tumors from ^68^Ga-FAPI-RGD PET/CT were approximately 3 times greater than those from ^18^F-FDG PET/CT (15.2 vs. 5.5, *P* < 0.001). Detailed data and representative images are presented in Table 1 and Figure 3, respectively.

**TABLE 1.** Comparison of SUV_max_ from ^68^Ga-FAPI-RGD and ^18^F-FDG PET/CT in Primary and Metastatic Tumors

Tumor type	*n*	Size (cm)	^68^Ga-FAPI-RGD PET/CT			^18^F-FDG PET/CT			*P* (FAPI-RGD vs. FDG)	
			Positive tumors (*n*)	SUV_max_	TBR	Positive tumors (*n*)	SUV_max_	TBR	Median SUV_max_	TBR
Primary										
Total	19	2.5 (0.6–6.5)	19	18.0 (7.0–40.3)	15.2 (7.0–57.6)	16	9.1 (1.7–15.5)	5.5 (1.7–14.3)	<0.001	<0.001
NPC	3	1.9 (1.3–2.4)	3	19.3 (16.5–25.8)	13.8 (10.3–15.2)	3	10.9 (7.8–11.8)	5.5 (4.9–7.9)	0.109	0.109
Breast cancer*	3	0.8 (0.6–0.8)	3	16.2 (7.1–18.9)	27.0 (11.8–31.5)	0	2.1 (1.7–2.4)	2.1 (1.7–2.4)	0.109	0.109
Esophageal cancer	4	2.4 (1.8–2.7)	4	30.1 (21.3–40.3)	33.4 (23.7–57.6)	4	12.1 (9.1–15.5)	7.2 (5.4–10.2)	0.068	0.068
NSCLC	3	2.6 (2.5–6.5)	3	14.8 (12.9–18.0)	16.1 (14.5–24.7)	3	7.8 (6.8–12.9)	10.4 (3.2–14.3)	0.18	0.109
SCLC	2	4.2 (3.6–4.8)	2	9.1 (8.9–9.2)	7.5 (6.1–8.9)	2	6.7 (5.5–7.8)	3.4 (3.2–3.5)	NA	NA
Pancreatic cancer	1	4.3 (NA)	1	36.3 (NA)	51.9 (NA)	1	13.8 (NA)	6.6 (NA)	NA	NA
Ovarian cancer	3	3.8 (3.2–4.5)	3	12.4 (7.0–25.6)	12.4 (7.0–14.2)	3	9.9 (8.1–12.6)	5.7 (5.0–6.8)	0.285	0.109
Metastases										
Lymph node mets (total)	129	1.1 (0.4–3.6)	128	12.1 (2.0–27.1)	13.3 (1.8–38.7)	117	6.1 (1.0–13.7)	4.1 (1.0–14.2)	<0.001	<0.001
Lung mets	25	0.9 (0.3–3.5)	19	8.0 (0.9–15.7)	15.6 (1.1–39.3)	17	4.9 (0.7–7.1)	10.0 (0.9–22.3)	<0.001	<0.001
Liver mets	14	1.4 (0.6–3.9)	14	11.2 (3.9–19.4)	7.3 (3.6–16.8)	11	5.3 (2.3–12.0)	1.9 (1.0–5.0)	0.005	0.001
Bone mets	80	1.3 (0.5–8.2)	80	16.2 (5.6–49.6)	15.6 (6.3–70.9)	64	5.2 (0.9–11.8)	4.7 (0.9–13.8)	<0.001	<0.001
Peritoneal mets	17	NA^†^	17	16.0 (6.4–18.8)	16.8 (3.6–22.9)	17	10.3 (4.7–14.1)	7.1 (2.6–10.1)	<0.001	<0.001
Other mets^‡^	13	0.8 (0.4–2.3)	13	8.6 (4.0–10.0)	10.0 (6.3–207.5)	9	6.6 (3.4–11.9)	3.4 (1.0–6.0)	0.196	0.001

* One patient was diagnosed with multifocal breast cancer (4 primary tumors).

† Lesion size cannot be calculated because of diffuse type of peritoneal metastasis (irregular shape).

‡ Other mets included splenic (*n* = 2), pleural (*n* = 1), adrenal (*n* = 1), brain (*n* = 4), and subcutaneous (*n* = 5) mets.

NPC = nasopharyngeal carcinoma; SCLC = small lung cancer; mets = metastases; NA = not applicable.

Continuous data are median and range. Quantitative data from ^68^Ga-FAPI-RGD and ^18^F-FDG PET/CT were acquired at 3 h and 1 h after injection, respectively.

**FIGURE 4.**
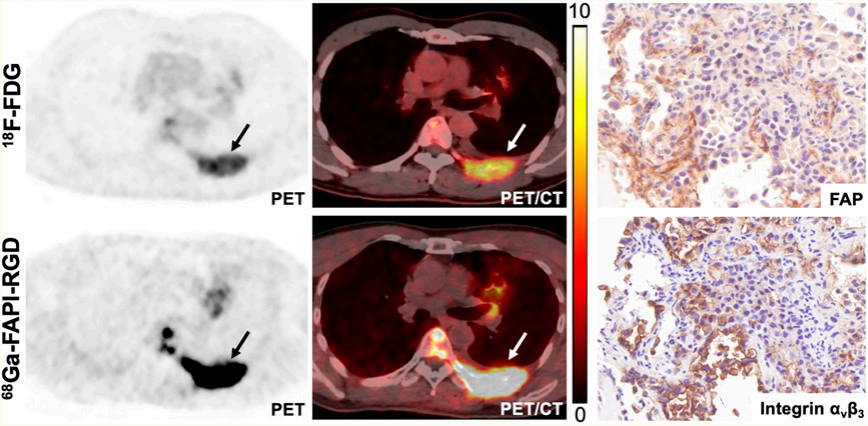
^68^Ga-FAPI-RGD and ^18^F-FDG PET/CT images in patient with metastatic lung adenocarcinoma. Immunohistochemical staining from left rib metastasis (arrow) revealed positive FAP and integrin α_v_β_3_ expression.

Among the 22 patients who underwent paired ^68^Ga-FAPI-RGD and ^18^F-FDG PET/CT, 129 lymph node metastases and 149 bone and visceral metastases were evaluated (including bone [*n* = 80], lung [*n* = 25], liver [*n* = 14], peritoneal [*n* = 17], and other [*n* = 13] metastases). ^68^Ga-FAPI-RGD PET/CT revealed a significantly higher SUV_max_ and TBR than did ^18^F-FDG PET/CT in most metastatic lesions. Interestingly, the SUV_max_ and TBR of lymph node metastases derived from ^68^Ga-FAPI-RGD PET/CT were 2 to 3 times greater than those derived from ^18^F-FDG PET/CT (SUV_max_, 12.1 vs. 6.1 [*P* < 0.001]; TBR, 13.3 vs. 4.1 [*P* < 0.001]), resulting in a greater number of metastatic lesions detected by ^68^Ga-FAPI-RGD than by ^18^F-FDG PET/CT (99% [128/129] vs. 91% [117/129], *P* = 0.003). Regarding imaging of bone and visceral metastases, ^68^Ga-FAPI-RGD PET/CT demonstrated significantly higher radiotracer uptake and a 2- to 3-fold higher TBR than did ^18^F-FDG PET/CT in most tumor lesions, including the lung, liver, bone, and peritoneal metastases. Consequently, ^68^Ga-FAPI-RGD PET/CT yielded significantly higher lesion detection rates than ^18^F-FDG for the diagnosis of bone and visceral metastases (96% [143/149] vs. 79% [118/149], *P* < 0.001). Representative PET/CT images with FAP and integrin α_v_β_3_ staining are presented in Figure 4.

### Comparison of ^68^Ga-FAP-RGD and ^68^Ga-FAPI-46 Uptake in Patients with Cancer

**FIGURE 5.**
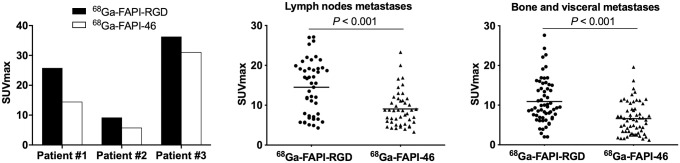
Quantitative comparison between ^68^Ga-FAPI-RGD and ^68^Ga-FAPI-46 in primary tumor (left), lymph node (middle), and bone and visceral metastases (right).

**FIGURE 6.**
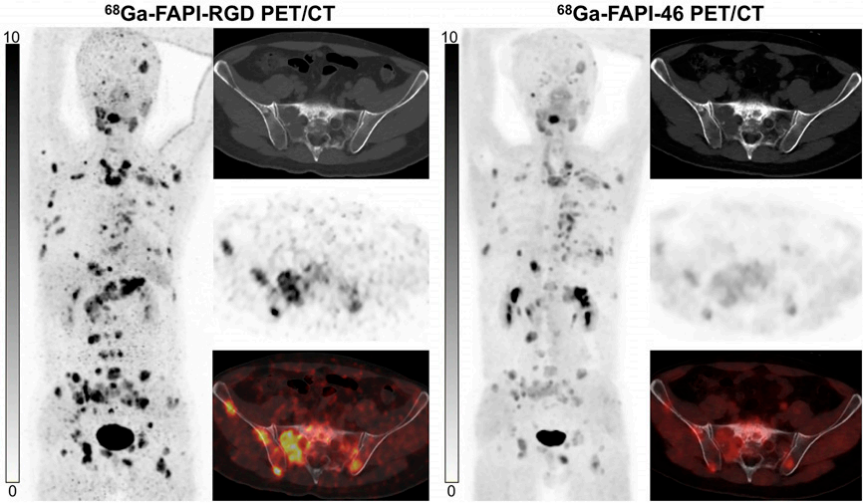
^68^Ga-FAPI-RGD PET/CT shows significantly higher radiotracer uptake than ^68^Ga-FAPI-46 in patient with metastatic thyroid cancer.

**FIGURE 7.**
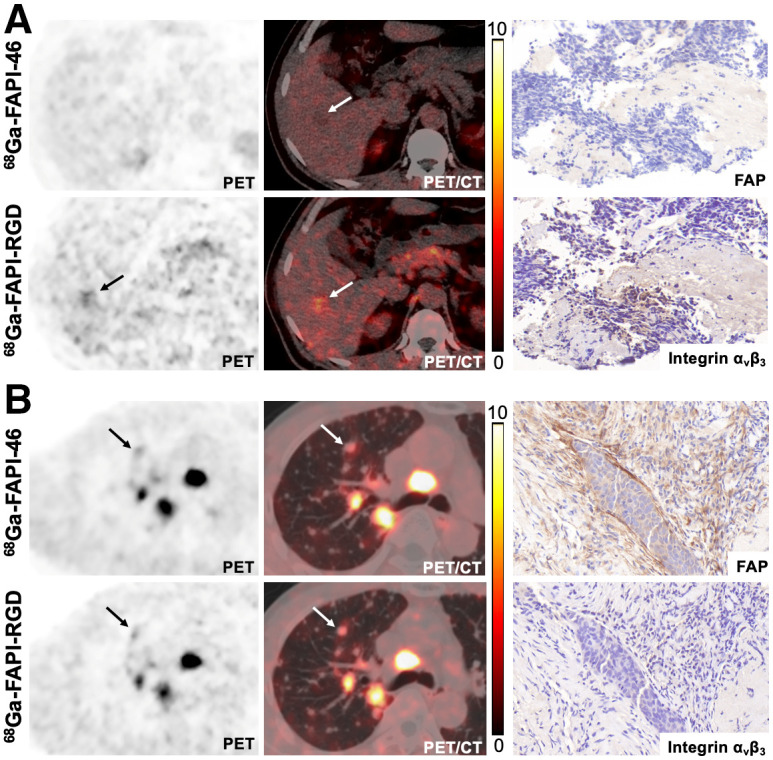
^68^Ga-FAPI-RGD and ^68^Ga-FAPI-46 PET/CT images and immunohistochemical staining in patients with metastatic small cell lung cancer (A) and nasopharyngeal carcinoma (B). Biopsy site is indicated by arrow.

^68^Ga-FAPI-RGD and ^68^Ga-FAPI-46 PET/CT were compared among 7 patients with 5 types of cancer. Three patients were treatment-naïve, and 4 had recurrent or progressive disease. Although the SUV_max_ and TBR of primary tumors from ^68^Ga-FAPI-RGD PET/CT seemed higher than those from ^68^Ga-FAPI-46 (SUV_max_, 25.8 vs. 14.5 [*P* = 0.109]; TBR, 15.2 vs. 7.6 [*P* = 0.109]), no statistical difference was observed (Table 2). Regarding the 102 metastatic lesions evaluated, ^68^Ga-FAPI-RGD PET/CT yielded a significantly higher SUV_max_ and TBR than did ^68^Ga-FAPI-46 in lymph node metastases (SUV_max_, 15.5 vs. 8.7 [*P* < 0.001]; TBR, 13.2 vs. 8.1 [*P* < 0.001]), lung metastases (SUV_max_, 9.5 vs. 6.4 [*P* = 0.004]; TBR, 18.8 vs. 12.5 [*P* = 0.003]), and bone metastases (SUV_max_, 13.7 vs. 6.8 [*P* < 0.001]; TBR, 14.5 vs. 6.3 [*P* < 0.001]) (Fig. 5). Representative PET images are presented in Figure 6. Interestingly, in 1 patient with small cell lung cancer, 4 bone metastases and 3 liver metastases exhibited no abnormal uptake of ^68^Ga-FAPI-46, but uptake of ^68^Ga-FAPI-RGD was increased. Immunohistochemical staining of a liver metastasis demonstrated negative FAP expression but positive integrin α_v_β_3_ expression (Fig. 7A). Notably, in another patient with nasopharyngeal carcinoma, both tracers exhibited similar uptake in several metastatic lung lesions, which demonstrated positive FAP expression and negative integrin α_v_β_3_ expression (Fig. 7B).

**TABLE 2.** Comparison of SUV_max_ on ^68^Ga-FAPI-RGD and ^68^Ga-FAPI-46 PET/CT Images in Primary and Metastatic Lesions

Tumor type	*n*	Tumor size (cm)	Tracer	Positive lesions (*n*)	SUV_max_		TBR	
					Median	*P*	Median	*P*
Primary tumors								
NPC	1	2.4 (NA)	^68^Ga-FAPI-RGD		25.8	NA	15.2	NA
			^68^Ga-FAPI-46		14.5		7.6	
SCLC	1	4.8 (NA)	^68^Ga-FAPI-RGD		9.2	NA	6.1	NA
			^68^Ga-FAPI-46		5.8		3.4	
Pancreatic cancer	1	4.3 (NA)	^68^Ga-FAPI-RGD		36.3	NA	51.9	NA
			^68^Ga-FAPI-46		31.1		38.9	
Total	3	4.3 (2.4–4.8)	^68^Ga-FAPI-RGD		25.8 (9.2–36.3)	0.109	15.2 (6.1–51.9)	0.109
			^68^Ga-FAPI-46		14.5 (5.8–31.1)		7.6 (3.4–38.9)	
Metastases								
Lymph node mets (total)	46	1.1 (0.6–2.8)	^68^Ga-FAPI-RGD	46	15.5 (4.3–27.1)	<0.001	13.2 (3.3–38.7)	<0.001
			^68^Ga-FAPI-46	46	8.7 (3.3–23.3)		8.1 (2.7–18.2)	
Brain mets	4	0.7 (0.4–0.7)	^68^Ga-FAPI-RGD	4	5.8 (4.0–8.3)	0.068	143.8 (80.0–207.5)	0.068
			^68^Ga-FAPI-46	4	3.0 (2.5–4.2)		83.7 (65.0–85.0)	
Lung mets	15	1.1 (0.5–1.4)	^68^Ga-FAPI-RGD	13	9.5 (2.0–15.7)	0.004	18.8 (2.0–39.3)	0.003
			^68^Ga-FAPI-46	13	6.4 (1.6–13.3)		12.5 (1.6–33.3)	
Liver mets	7	1.1 (0.6–1.4)	^68^Ga-FAPI-RGD	7	6.9 (3.9–16.8)	0.028	6.3 (3.6–16.8)	0.028
			^68^Ga-FAPI-46	4	3.4 (1.8–16.2)		3.4 (1.8–16.2)	
Subcutaneous mets	5	0.8 (0.7–0.9)	^68^Ga-FAPI-RGD	5	8.6 (6.6–10.0)	0.138	7.2 (5.5–10.0)	0.08
			^68^Ga-FAPI-46	5	7.2 (6.1–9.0)		5.1 (3.6–8.2)	
Bone mets	29	1.2 (0.5–8.2)	^68^Ga-FAPI-RGD	29	13.7 (6.1–27.6)	<0.001	14.5 (6.3–27.6)	<0.001
			^68^Ga-FAPI-46	25	6.8 (1.2–19.6)		6.3 (1.8–21.8)	

NPC = nasopharyngeal carcinoma; NA = not applicable; SCLC = small lung cancer; mets = metastases.

Data in parentheses are ranges.

## DISCUSSION

As pan-cancer biomarkers, FAP with radiolabeled FAPI-based molecules (including FAPI-04/46) have yielded encouraging results for PET imaging of cancer (7*,*^9^). However, the relatively short tumor retention time may hamper the use of FAP molecules for radioligand therapy applications. We and others have applied the polyvalence effect to develop homomultimers to enhance tumor uptake and retention, and dimeric FAPI-based tracers have been synthesized and evaluated (17*,*^18^). A dual-receptor–targeting approach with a heterodimer is another strategy to improve the tumor-targeting efficacy, especially for imaging probes that recognize only 1 receptor (19). Considering that our previous data showed tumor uptake and retention of ^68^Ga-FAPI-RGD to be significantly greater than those of ^68^Ga-FAPI-46 and ^68^Ga-c(RGDfK) in mouse xenografts (10), we speculated that ^68^Ga-FAPI-RGD would be a promising radiotracer for imaging tumors expressing either FAP or integrin α_v_β_3_. In the present study, ^68^Ga-FAPI-RGD, a heterodimeric PET tracer that targets both FAP and integrin α_v_β_3_, was evaluated in 3 healthy volunteers and 22 patients with cancer. Our clinical studies demonstrated ^68^Ga-FAPI-RGD to be a promising PET agent that allows imaging of various types of cancer. Its dual-receptor–targeting property results in improved tumor uptake and retention, allowing imaging of tumors with either or both receptor expression patterns.

^68^Ga-FAPI-RGD was safe and well tolerated in all healthy volunteers and patients with cancer. The average effective whole-body dose of ^68^Ga-FAPI-RGD was 1.01 × 10^−2^ mSv/MBq, which is comparable to the effective doses of ^68^Ga-FAPI-02 and ^68^Ga-FAPI-04 (1.64 × 10^−2^ mSv/MBq and 1.80 × 10^−2^ mSv/MBq) (16). However, intense physiologic ^68^Ga-FAPI-RGD uptake was observed in the thyroid and pancreas, with a distribution pattern similar to that of ^68^Ga-FAPI dimers previously reported by us and others (17*,*^18^). Interestingly, significantly decreased ^68^Ga-FAPI-RGD activity was observed in normal organs (particularly in the thyroid, pancreas, and salivary glands), whereas increased uptake was observed in tumor lesions from 0.5 to 3 h after injection, resulting in optimized lesion contrast in delayed scans. Therefore, we speculate that additional delayed ^68^Ga-FAPI-RGD PET/CT scans may help improve the lesion detection rate and offer advantages for discrimination of tumor and nontumor lesions. However, the clinical benefits of delayed scans require further investigation in a larger patient population.

The SUV_max_ and TBR of primary tumors from ^68^Ga-FAPI-RGD PET/CT were significantly higher than those from ^18^F-FDG PET/CT, particularly in non–small cell lung cancer (NSCLC) and in esophageal, breast, and pancreatic cancers. The reason for this finding is that all primary tumors (19/19) were satisfactorily visualized via ^68^Ga-FAPI-RGD, whereas 3 breast cancer lesions were missed via ^18^F-FDG. ^68^Ga-FAPI-RGD PET/CT also demonstrated significantly greater radiotracer uptake in lymph node, bone, and visceral metastases and a significantly higher TBR than ^18^F-FDG PET/CT, resulting in an improved lesion detection rate, particularly for the diagnosis of lymph node (99% vs. 91%) and bone (100% vs. 80%) metastases. However, in our previous ^68^Ga-FAPI-RGD PET/CT study on 6 patients, the tumor uptake of ^68^Ga-FAPI-RGD did not differ from that of ^18^F-FDG (10). Possible explanations may be the limited number of patients, different cancer types investigated, and different acquisition time after injection (30–120 min vs. 60–180 min). According to the results of the present study, ^68^Ga-FAPI-RGD may be more suitable for imaging tumors with both FAP and integrin α_v_β_3_ expression, particularly for NSCLC and esophageal cancer (20*,*^21^). First, the radiotracer uptake and TBR derived from ^68^Ga-FAPI-RGD PET/CT were higher than those from ^18^F-FDG in these cancer types, resulting in improved lesion detectability, particularly of liver, bone, and brain metastases. These results suggest that ^68^Ga-FAPI-RGD PET/CT may contribute to the diagnosis of NSCLC and esophageal cancer, especially in detecting small metastases with low-to-moderate uptake on ^18^F-FDG PET/CT. Second, false-positive findings in the mediastinal lymph nodes often confound interpretation of preoperative ^18^F-FDG PET/CT images in NSCLC and esophageal cancer. On the basis of previous publications, nonmetastatic reactive lymph nodes presenting increased ^18^F-FDG uptake might be correctly diagnosed either by FAP or by integrin α_v_β_3_–targeting radiotracers (21–24). Therefore, ^68^Ga-FAPI-RGD PET/CT may be more suitable than ^18^F-FDG PET/CT for determining the preoperative lymph node status in these cancer types. Taken together, the results indicate that NSCLC and esophageal cancer may be potential indications for future clinical use of ^68^Ga-FAPI-RGD, and further prospective studies are warranted to confirm this possibility.

PET imaging demonstrated that the radiotracer uptake and TBR of ^68^Ga-FAPI-RGD were significantly higher than those of ^68^Ga-FAPI-46, especially in the involved lymph node, bone, and visceral metastases. Interestingly, we noted that several lesions from metastatic small cell lung cancer and thyroid cancer exhibited low uptake of ^68^Ga-FAPI-46 but higher uptake of ^68^Ga-FAPI-RGD. Histopathologic results revealed low expression of FAP but higher expression of integrin α_v_β_3_. Therefore, ^68^Ga-FAPI-RGD PET/CT may be used to image tumors that are either FAP-positive or integrin α_v_β_3_–positive, whereas ^68^Ga-FAPI-46 failed to visualize lesions that were FAP-negative and integrin α_v_β_3_–positive. Moreover, both tracers showed similar uptake in lesions with FAP-positive and integrin α_v_β_3_–negative expression, suggesting comparable FAP-targeting ability between ^68^Ga-FAPI-RGD and ^68^Ga-FAPI-46. On the basis of these findings, we speculate that ^68^Ga-FAPI-RGD PET/CT would be superior to ^68^Ga-FAPI-46 PET/CT for the diagnosis of cancer, especially when ^68^Ga-FAPI-46 PET/CT findings are inconclusive.

With the rather rapid washout from tumors and unsatisfactory therapeutic efficacy, ^177^Lu-FAPI-04/46 may not be an optimal targeting vector for radioligand therapy (25*,*^26^). Besides the improved tumor uptake of ^68^Ga-FAPI-RGD, the prolonged tumor retention was an unexpected finding in this study, as may be explained by the synergistic interaction between the 2 binding motifs in the heterodimer. It is possible that the binding of the first motif, even if only temporary, may first direct ^68^Ga-FAPI-RGD to the target surface or reduce the off-rate of ^68^Ga-FAPI-RGD, allowing the second binding motif to also attach to the tumor and therefore increasing the overall binding and the probability of adhering to the tumor (27). Improved tumor accumulation and prolonged tumor retention are the potential advantages of FAPI-RGD over the current FAPI variants, making it a suitable targeting vector after labeling with ^177^Lu/^90^Y/^225^Ac for therapeutic applications. In addition, the rapid clearance of ^68^Ga-FAPI-RGD in normal organs, particularly in the thyroid, salivary glands, and blood pool, may decrease the absorbed dose delivered to normal human tissues. This possibility should be extensively studied in nuclear oncology research in the future.

Our study had several limitations. First, because of the relatively short half-life of ^68^Ga, the in vivo distribution pattern and tumor retention of FAPI-RGD could not be fully investigated. Second, because few patients underwent paired ^68^Ga-FAPI-RGD/^18^F-FDG (*n* = 22) or ^68^Ga-FAPI-RGD/^68^Ga-FAPI-46 PET/CT imaging (*n* = 7), only a descriptive comparison was possible. Third, this study focused primarily on lesion detection rates, and the specificity of ^68^Ga-FAPI-RGD PET/CT requires further investigations with histopathologic confirmation. Furthermore, we were unable to compare ^68^Ga-FAPI-RGD PET/CT with ^68^Ga-RGD PET/CT in the same group of patients because of ethical considerations. ^68^Ga-FAPI-RGD and ^68^Ga-RGD PET/CT should be compared in future clinical research.

## CONCLUSION

The study demonstrated the safety and clinical feasibility of ^68^Ga-FAPI-RGD PET/CT for imaging of various types of cancer. Further investigation of the diagnostic accuracy of ^68^Ga-FAPI-RGD as a PET tracer, as well as its antitumor efficacy after labeling with therapeutic radionuclides, is necessary.

## DISCLOSURE

This work was funded by the National Natural Science Foundation of China (82071961), Key Scientific Research Program for Young Scholars in Fujian (2021ZQNZD 016), Fujian Natural Science Foundation for Distinguished Young Scholars (2022J01310623), Fujian Research and Training Grants for Young and Middle-aged Leaders in Healthcare, Key Medical and Health Projects in Xiamen (3502Z20209002), The National University of Singapore Start-up Grant (NUHSRO/2020/133/Startup/08), NUS School of Medicine Nanomedicine Translational Research Programme (NUHSRO/2021/034/TRP/09/Nanomedicine), and National Medical Research Council (NMRC) Centre Grant Programme (CG21APR1005). Liang Zhao was partially funded by the China Scholarship Council (CSC). No other potential conflict of interest relevant to this article was reported.

## Notes

KEY POINTS **QUESTION:** Will a heterodimer that recognizes both FAP and integrin α_v_β_3_ yield improved tumor uptake compared with ^18^F-FDG or ^68^Ga-FAPI-46? **PERTINENT FINDINGS:** This clinical study revealed that the tumor uptake and TBR of ^68^Ga-FAPI-RGD were significantly higher than those of either ^18^F-FDG or ^68^Ga-FAPI-46 in various types of cancer. **IMPLICATIONS FOR PATIENT CARE:** The dual-receptor–targeting property of ^68^Ga-FAPI-RGD results in improved tumor uptake compared with ^18^F-FDG or ^68^Ga-FAPI-46, enabling imaging of tumors with either or both receptor expression patterns.
